# Integrating Flow Field Dynamics and Chemical Atmosphere Predictions for Enhanced Sulfur Corrosion Risk Assessment in Power Boilers

**DOI:** 10.3390/ma17194919

**Published:** 2024-10-08

**Authors:** Dariusz Kardaś, Sylwia Polesek-Karczewska, Izabela Wardach-Świȩcicka

**Affiliations:** Institute of Fluid-Flow Machinery, Polish Academy of Sciences, 80-231 Gdańsk, Poland

**Keywords:** CFD analysis, sulfur corrosion, criterion of parameters’ variance, mixture fraction, flow field fluctuations

## Abstract

In this work, we attempt to explain the phenomenon of sulfur corrosion of power boiler water walls under the conditions of large fluctuations in carbon monoxide concentrations. To assess the conditions required for corrosion formation, a criterion based on the chemical and flow field parameters of the flue gas is proposed. The formulated sulfur corrosion criterion is based on the mixture fraction variance and the turbulence time scale. Numerical modeling of coal combustion in a 250 MW power boiler is performed using ANSYS. Two cases of combustion in a boiler are analyzed, with the first simulating the boiler operated using classic high-swirl burners and the second one accounting for boiler operation with modified low-swirl burners. Calculations of pulverized coal combustion are performed using the standard k-ε turbulence model and the combustion described by the mixture fraction. The simulation results reveal that the low-swirl burner is characterized by higher values of the mixture fraction variance and a higher frequency of fluctuation of the velocity field, which is strongly related to an increased corrosion rate. The study outcomes show the validity of using the criterion of the mixture fraction variance and velocity field fluctuations to determine the areas at risk of sulfur corrosion.

## 1. Introduction

The energy industry in Europe is undergoing rapid changes that could lead to a new structure based on renewable energy sources. Despite the rapid development of wind energy and photovoltaics, most of our energy and electricity is currently produced using conventional fuels. As forecasted by the International Energy Agency [1], coal will continue to have a prominent place in the energy sector in many countries. Over the coming decades, the share of fossil fuel-based power generation will be significant, if only because in China and India, a very large proportion of coal-fired power plants are new. This prospect highlights the need to adapt coal-fired installations to stringent environmental requirements [2,3,4].

Corrosion process development in boilers and furnaces is rooted in several factors, including fuel chemical composition, flue gas and tubes’ surface temperature, flue gas composition, and properties of ash deposits on the heat-transfer surfaces. For this reason, corrosion risk has been and still is a significant technical issue in all devices that burn solid fuels. In particular, high-temperature corrosion has been the subject of extensive studies over the past few decades. It involves not only coal-fired systems, but also those that utilize other types of renewable fuels, such as various kinds of forest, agricultural, and waste biomass [5,6,7] that have become favorable due to green energy development trends. Therefore, expertise is needed to adapt existing or design new energy-generation units to use alternative biogenic fuels, including also scenarios that involve co-firing with coal. In this respect, the alkali- and chlorine compounds in biomass are pf real concern [8,9], as they are accepted as key high-temperature corrosion-inducing factors through forming salts on the heat transfer surfaces. To address the issue of deposit formation, the cited research involves a detailed study of the release characteristics and transformation pathways of these harmful species [10,11,12]. Alkali sulfates have a less negative impact on the corrosion rate than alkali chlorides, owing to their higher melting points. High-temperature corrosion mitigation is thus considered in terms of sulfation control for the fuel type utilized [8].

Since corrosion in large-scale boilers largely contributes to increased maintenance costs and adversely affects the operation reliability and safety of power plant units, efforts are made to monitor corrosion rates and predict the process paths to prevent its development and, thereby reduce its negative effects [13,14]. A substantial number (nearly 50%) of heating surface failures in power boilers and the reason for their necessary shutdowns are associated with a decrease in heat transfer rates and with superheaters sustaining damage due to the accumulation of chemically active ash [15,16].

To analyze the high-temperature corrosion behavior of austenitic heat-resistant steel under a real flue gas atmosphere, Liang et al. [17] examined the tubes cut from final re-heaters in a 660 MW ultra-supercritical power plant. Based on the detailed morphological and chemical analyses of deposits on the sample tubes, the authors proposed the mechanism of high-temperature corrosion. Likewise, Yu et al. [18] studied the high-temperature sulfide-type corrosion mechanism based on a detailed laboratory analysis of the tube samples cut down from the 300 MW coal-fired boiler. Their research included the physicochemical properties of the corrosion products in different sample layers in terms of their chemical composition, mineralogy, and morphology. Allgurén et al. [8] focused on analyzing sulfation process effectiveness with regard to alkali-related corrosion in co-firing. The obtained results indicated the crucial impact of sulfur–alkali and chlorine–alkali ratios on the formation of alkali sulfates and alkali chlorides.

Sanusi et al. [19] investigated the fireside corrosion rates for waste wood and herbaceous grass combustion through the experimental furnace tests on two heat-exchanger steels exposed to deposits with various compositions. The study was performed using sample tubes coated with the deposits and in the gas environment, both defined and prepared based on the thermodynamic equilibrium simulations. Dai et al. [20] performed an analysis of high-temperature ash-induced tube corrosion for the selected Pakistani lignite coals. Through long-term corrosion tests (up to 200 h) on low-alloy steel in the bench-scale facility under the typical firing conditions in an air atmosphere at 650 °C, the researchers determined the kinetics of the tube oxidation. Recently, Kaniowski et al. [21] investigated the corrosion resistance of boiler steels and alloy coatings subjected to aggressive ash deposits under high-temperature conditions. Their research was focused on examining the surface of steel samples covered with various types of ash after long-term heating (for 4 months) in a laboratory furnace at 750 °C under oxidizing conditions. Furugaki et al. [22] performed ash-embedded corrosion tests of heat-resistant steels using combustion ash collected from an actual super-heater tube to examine the effect of the temperature gradient of ash on the high-temperature corrosion behavior of steels. Maj et al. [23] investigated high-temperature corrosion taking place in a full-scale multifuel coal fluidized bed (CFB) boiler, based on the analysis of corrosion products originating from the boiler. In addition, through the long-term laboratory corrosion tests of waterwall steel samples under ash deposits, the authors determined the process rate.

The problem of high-temperature corrosion in boilers has intensified with the implementation of low-emission combustion technologies targeted at reducing NOx emissions [24]. It has been observed that the use of low-NOx burners and air staging, which are the key elements of this technology, leads to oxygen deficiency and excess of carbon monoxide in the area near the screen tube walls [25]. Studies focusing on corrosion in the furnaces has, therefore, focused on identifying the zones of reducing atmosphere. Xiong et al. [26] analyzed high-temperature sulfur corrosion in a pulverized coal subcritical 300 MW furnace based on the sampling and characterization of cut-down corroded tube samples and the synchronized measurements of reducing gas atmosphere (including CO, $O_{2}$, $H_{2}S$ and $H_{2}$) in the vicinity of the water wall tubes. Li et al. [27] studied the $H_{2}S$ formation and evolution in the reduction zone of pulverized coal air-staged combustion. They developed and implemented a sulfur release model and validated the simulation results with experimental data performed for 17 different coal types using a drop tube furnace.

Liu et al. [28] investigated the effect of air distribution on high-temperature corrosion in the 350 MW subcritical pulverized coal boiler via online monitoring of the $H_{2}S$ fluctuations and the volume fractions of CO and $O_{2}$ in the furnace. There are also studies that utilized Computational Fluid Dynamics (CFDs) to assess the high-temperature corrosion risk. Modlinski and Hardy [14] carried out three-dimensional (3D) CFDs simulations for 160 MW industrial pulverized coal boiler implementing four global gas phase kinetic mechanisms to predict the CO and $O_{2}$ concentrations in near-water walls zones. To verify the simulation results, the authors compared them with the measurement data derived from the online monitoring system and obtained satisfactory qualitative agreement in terms of $O_{2}$ concentration. Liu et al. [29] conducted a numerical evaluation of the impact of the near-wall air position on reducing atmosphere, combustion, and nitrogen oxide emissions in a 1000 MW opposed wall-fired boiler. Recently, Jin et al. [30] numerically investigated a staged combustion in a 1000 MW double-tangential circular coal-fired boiler with separated overfire air distribution, paying special attention to reducing atmosphere near the water walls of the upper furnace to mitigate the corrosion hazard. Similarly, Zhong et al. [31] studied the effect of air distribution on boiler slagging and high-temperature corrosion of a 1000 MW ultra-supercritical double tangentially fired boiler through numerical simulation, while implementing an optimized $H_{2}S$ reaction mechanism. In other work, Jin et al. [32], performed CFDs simulations to analyze the effect of primary air ratios on the combustion behavior of a 1050 MW double-tangential coal-fired boiler under ultra-low load while considering the corrosion hazard, in addition to the issues of combustion stability, coal burnout and NOx emissions. Their study identified the areas around the operating coal burners to be of highly reducing atmosphere and thereby at increased risk of high-temperature corrosion. Von Bohnstein and co-workers [33] carried out 3D CFDs simulations of an entrained flow reactor for coal combustion to predict gaseous compounds contributing to corrosion development. By implementing the detailed coal combustion chemistry, the authors analyzed the concentrations of CO and $O_{2}$ in the flow reactor and arrived at predicted concentrations of corrosive sulfur and chlorine species, including ${\mathrm{SO}}_{2}$, $H_{2}S$, COS, and HCl—results which were consistent with measured values.

The problem of high-temperature corrosion has also appeared at a large scale in pulverized coal boilers operated in Polish CHP plants when low NOx combustion was introduced [34]. This involves, among others, the sulfur corrosion of the large-scale OP-230 type steam boiler, which is the objective of the present study. This is a 250 MW capacity boiler with six pulverized coal burners. The problems with corrosion arose when new burners with low NOx emission levels were used instead of the typical high-swirl burners. The real-scale measurements carried out indicated that when the latter were used, the maximum rate of material loss of the tubes’ wall was about 0.09 mm/year. After new burners were installed, the rate of corrosion increased by 8 times.

The literature review shows that the main focus in the current research on high-temperature corrosion is either on recognizing the corrosive properties of ash deposits or the combustion chemistry to determine the concentrations of the corrosion-inducing flue gas components in the vicinity of the water wall tubes. To our knowledge, there is a lack of studies in this field that allow the flow parameters to be directly related to the corrosion rate assessment. The purpose of this work is to contribute to a better understanding of high-temperature sulfur corrosion by defining the flow and chemical conditions that promote tube degradation in a boiler. A novel approach is developed, which utilises computational fluid dynamics to analyze the complex process of corrosion that occurs at the microscale level. Based on the mixture fraction method that simplifies the tracking of species transport by reducing it to a single scalar, the criterion is proposed, linking mixture fraction variance and velocity field fluctuations. Considering the complex chemical reaction mechanism involved in the process, such a method allows us to shorten the computational time needed to solve all necessary transport equations for each chemical element involved in each single-step chemical reaction. The proposed corrosion index proved to correspond well with the corrosion rate derived based on long-term measurements in a large-scale pulverized coal boiler, offering a reliable and efficient option for corrosion hazard assessment and prevention.

## 2. Model of Corrosion

Several models have been developed to understand and predict sulfur corrosion in high-temperature environments, particularly in applications like oil refining, gas processing, and coal combustion. The most well-known models are based on Wagner’s oxidation theory of high-temperature sulfidation, in which sulfur reacts with metal surfaces to form metal sulfides [35,36]. Wagner’s theory explains the growth of sulfide scales on metals, considering the diffusion of metal cations and sulfur anions through the sulfide layer. It is primarily used for pure metals and has been simplified for binary systems. It does not account for more complex environments, such as the presence of other corrosive species (e.g., oxygen or chlorides).

The models useful for high-temperature combustion environments (e.g., coal combustion, gas turbines), in which both oxygen and sulfur are present, are called Mixed Oxide–Sulfide Layer models [37]. Those models expand upon Wagner’s theory by considering mixed layers of oxides and sulfides on metal surfaces exposed to an atmosphere containing oxygen and sulfur. These species compete to form their respective compounds (metal sulfides and oxides) depending on local temperature and their partial pressures. The interaction between multiple species (e.g., sulfur, oxygen, and chlorine) can complicate the predictions.

Other groups of models are based on the thermodynamic equilibrium principle [38,39]. They can predict the thermodynamically stable phases (sulfides, oxides, and other compounds) that form under specific conditions. They are based on Gibbs free energy minimization and calculate the equilibrium between the sulfur-containing gas phase species (e.g., $H_{2}S$, ${\mathrm{SO}}_{2}$) and the metal surface at various temperatures and pressures. This assumption makes them quite fast (depending on the number of species and compounds) and allows them to be widely used for material selection and process design in high-sulfur environments, but they do not cover the dynamics predictions of the processes. They can predict only equilibrium states, not the actual kinetics or rates of corrosion, which are often influenced by diffusion and other factors.

More complex models, which are useful for understanding time-dependent corrosion, especially in high-temperature equipment like furnaces and heat exchangers, are the so-called Kinetic Models, which consider both thermodynamics and the kinetics of the corrosion [40,41]. They are capable of predicting the rate of corrosion resulting from a sulfur attack and they incorporate parameters like diffusion rates of sulfur species, temperature, and the thickness of protective layers. However, the application of these models may be limited to some extent due to the need for detailed kinetic data and real-time measurements. They enable a reduction in the number of species and reactions, but there is a need to determine which of the considered reactions is the more important.

Furthermore, Sulfur-Induced Stress Corrosion Cracking (SCC) models explain cracking due to the combined effects of mechanical stress and sulfur corrosion [42,43]. The main assumption is that sulfur, particularly in the form of $H_{2}S$, can weaken metals by forming metal sulfides, which lead to embrittlement and stress cracking. This type of modelling is particularly important in the oil and gas industry, where materials are exposed to $H_{2}S$-rich environments. They are often dependent on specific material properties and environmental factors like stress levels, so results can vary widely.

Each group of models has specific applications depending on whether the investigation is targeted at predicting the extent or the rate of corrosion or at understanding material behavior under combined mechanical and chemical stress. Nevertheless, they are focused on predicting the detailed processes that mostly occur in a solid–fluid interaction zone.

It is difficult to find a common uniform view of the high-temperature sulfur corrosion mechanism, and there is no standard description of the phenomenon [21]. A consensus, however, has been reached regarding the root causes of corrosion. The name *sulfur corrosion* itself indicates that it is the presence of sulfur in coal that largely contributes to the corrosion process. The degradation of screen tubes occurs as a result of chemical reactions and the melting of deposits on their outer walls.

In the analysis, the following chemical reactions are considered:

- Iron oxidation
  (1) $$4\mathrm{Fe}+3O_{2}\to 2{\mathrm{Fe}}_{2}O_{3}\;,$$
- Iron reduction
  (2) $$2{\mathrm{Fe}}_{2}O_{3}+9C+4{\mathrm{SO}}_{3}\to 4\mathrm{FeS}+9{\mathrm{CO}}_{2}\;,$$
- Oxidation of sulfur dioxide
  (3) $$2{\mathrm{SO}}_{2}+O_{2}\to 2{\mathrm{SO}}_{3}\;,$$
- Carbon dioxide regeneration
  (4) $$2\mathrm{CO}\to {\mathrm{CO}}_{2}+C\;.$$

Composing the reactions presented above leads to the following global reaction:

(5) $$4\mathrm{Fe}+5O_{2}+18\mathrm{CO}+4{\mathrm{SO}}_{2}\to 4\mathrm{FeS}+18{\mathrm{CO}}_{2}\;,$$

which defines the impact of oxygen, carbon oxide and sulfur dioxide.

Another factor that affects the course of corrosion is the liquified form of deposits on the boiler tube wall. Observations of dust agglomeration on the screen pipe show [44] that the deposits are thicker on the leeward tube side, where the flow separation occurs. Hence, the temperature of deposits on this pipe’s side will be higher as the heat transfer deteriorates. Another important factor in corrosion is the variation in flue gas chemistry. Where conditions remain unchanged, corrosion is slow, even though the flue gas has a strong reducing effect on iron oxides. Corrosion is favored by fluctuations in ${\mathrm{SO}}_{2}$, CO and $O_{2}$ concentrations at temperatures above the melting point of the deposits [45]. The pipes’ weight loss is caused by mechanical factors when oxidation and reduction products lose their cohesiveness with the pipe material. In a single oxidation–reduction cycle, the weight loss of iron denoted by $\Delta m_{Fe}$ at unit area *A* depends on the reaction rate constant at the pipe surface $k_{c}$, the temperature of the deposit on the wall $T_{d}$, the melting point of deposit $T_{m}$, the concentration of ${\mathrm{SO}}_{2}$, CO and $O_{2}$, which may be described by the global reaction:

(6) $${\left(\frac{\Delta m_{Fe}}{\Delta A}\right)}_{cyc}=-k_{c}\exp\left(-\frac{T_{m}}{T_{d}}\right){\left[{\mathrm{SO}}_{2}\right]}_{ox}^{a}{\left[O_{2}\right]}_{ox}^{b}{\left[\mathrm{CO}\right]}_{r}^{c}\;.$$

The parameters *a*, *b*, and *c*, representing the stoichiometric coefficients for the respective species, are determined based on the global reaction Equation (5) while referring to the single Fe compound. This yields the values of a=1, b=5/4, c=18/4. Subscripts ox and *r* refer to oxidation and reduction conditions, respectively.

Further, it may be assumed that the surface processes are very fast and result in the pipe being covered with oxide almost immediately. The reduction reaction is just as fast. It should be noted that the concentrations of the individual components in Equation (6) are not the average values, but the quantities that correspond with the respective phase of a cycle. Corrosion occurs on the tube’s surface but depends on the processes taking place in the gas phase; thus, the condition for the reduction of iron oxide is its prior formation in the oxidation reaction. Under such assumptions, the mass of corroded iron is a function of the duration of one cycle $\Delta t_{cyc}$. The shorter the cycle time, the faster the corrosion, according to the following equation:

(7) $$\frac{1}{\Delta A}\frac{dm_{Fe}}{dt}=\frac{1}{\Delta t_{cyc}}{\left(\frac{\Delta m_{Fe}}{\Delta A}\right)}_{cyc}=-\frac{k_{c}}{\Delta t_{cyc}}\exp\left(-\frac{T_{m}}{T_{d}}\right){\left[{\mathrm{SO}}_{2}\right]}_{ox}{\left[O_{2}\right]}_{ox}^{5/4}{\left[\mathrm{CO}\right]}_{r}^{18/4}\;.$$

It should be noted that the practical use of the above formula is a problematic issue because the corrosion rate constant $k_{c}$ depends on the material and the chemical composition of the deposits on a pipe surface. Corrosion reactions occur at the scale of atoms on the screen pipes’ surface, but the corrosion sources are in the surrounding flue gas components. This fact forms the basis of the proposed approach to determining the ability of the exhaust gas to form corrosion.

## 3. Novel CFDs-Based Approach

A new concept for assessing sulfur corrosion in a boiler is to ignore phenomena in the pipe material and account for the variability and composition of the flue gas around the screen tubes. In this approach, instead of calculating the corrosion rate that directly involves the chemical reactions with the tube material according to Equation (7), the analysis is focused on the estimation of the flue gas’s capability to form corrosion. This quantity (parameter) is supposed to show whether the exhaust gas composition is conducive to sulfur corrosion.

A tool for determining the sulfur-induced corrosivity index of the flue gas could be the programs used to calculate the combustion process using the mixture fraction *f* and the variance of a mixture fraction ${f^{\prime }}^{2}$. The computational fluid dynamics (CFDs) software allows us to calculate the composition of the flue gas and fluctuations in a flue gas composition, as well as the turbulent velocity field of the gas flows. Using turbulence models and the probability distribution density function of the mixture fraction *f* makes it possible to determine the quantity that characterises the corrosiveness of flue gas.

In the study on high-temperature sulfur corrosion in the considered pulverized coal furnace, as demonstrated here, the mixture fraction method was used to model the combustion process [46]. Assuming equal diffusivities of all components, the species equations could be reduced to a single equation for the mixture fraction. In this case, instead of solving multiple transport equations, one equation is solved. The mixture fraction *f* itself is the mass fraction originating from the fuel stream, which means that for the fuel, the mixing degree is 1 and for the oxidant, it is 0. In addition to the mixture fraction, the combustion process is described by the transport equation for the mixture fraction variance ${f^{\prime }}^{2}$, which is a measure of the variability of the mixture fraction. The greater the variance, the greater the composition variability of the exhaust gases.

In general, the chemical reaction times are significantly shorter than the time scales of flow processes. In further considerations, the frequency of the process ω, determined by the inverse of the process time Δt, is taken into account. Regarding the cycles of iron oxidation and reduction, the frequency of chemical composition changes is limited by the frequency of velocity field fluctuations, and thus the following condition occurs

(8) $$\omega_{chem}\leq \omega_{vel}.$$

The latter, represented by $\omega_{vel}$, may be determined based on the two-equation k−ε turbulence model, wherein it is defined as follows:

(9) $$\omega_{vel}=\frac{k}{\varepsilon }\;,$$

where *k* denotes the turbulence kinetic energy (KTE) and ε is the KTE dissipation rate.

The fluctuation frequency of redox (corrosion) chemical reactions ($\omega_{chem}$) is limited by fluctuations in turbulent flows, hence

(10) $$\omega_{chem}=\frac{1}{\Delta t_{cyc}}\leq \omega_{vel}\;.$$

Considering Equation (7), one may observe that the corrosion rate depends on the concentrations of ${\mathrm{SO}}_{2}$ and $O_{2}$ in the oxidation phase and on the CO concentration in the reduction phase. It is noteworthy that these phases represent different stages of a corrosion process, differing in the flue gas composition. It is therefore assumed that a measure of the ability of the exhaust gas to induce corrosion is the variation in its chemical composition in the zone close to the water-wall tube panels. According to observations of sulfur corrosion, this is favored by the presence of carbon monoxide, but in a variable reducing-oxidizing atmosphere [45].

Taking into account Equations (7)–(10), and the fact that the concentration of ${\mathrm{SO}}_{2}$ in the flue gas is of the order of ppm, much smaller compared to CO concentration (approx. 5%), a parameter called the *Corrosion Index* is introduced, and is expressed as

(11) $$CI=\omega_{vel}\exp\left(-\frac{T_{m}}{T}\right){f^{\prime }}^{2}{\left[\mathrm{CO}\right]}^{18/4}\;.$$

It shall be noted that following Equation (7), the concentration of ${\mathrm{SO}}_{2}$ at the level of ppm disables the calculation of the corrosion rate due to the corrosion rate value tending to zero, regardless of the values of other parameters. In addition, a<b<<c. This allows us to neglect the ${\mathrm{SO}}_{2}$ and $O_{2}$ concentrations in the considered formula.

The proposed parameter characterises the probability of corrosion and is a function of the frequency of turbulent flow field, flue gas temperature (*T*), the variance of a mixing degree (${f^{\prime }}^{2}$) and CO concentration. It is expected that the probability of corrosion is larger in zones of higher fluctuations in flue gas composition at a high fluctuation frequency of an average flow field. On the other hand, the function described by Equation (11) decreases noticeably for flue gas at temperatures lower than the melting temperature of the deposits. During the sulfur corrosion process, the CO concentration plays a crucial role, as confirmed by the study of Jin et al. [32], among others. The CI factor, in the form proposed, may be used not as an absolute value, but as a parameter that compares the corrosive environments in various boiler configurations.

## 4. Simulation Results

**Figure 1:**
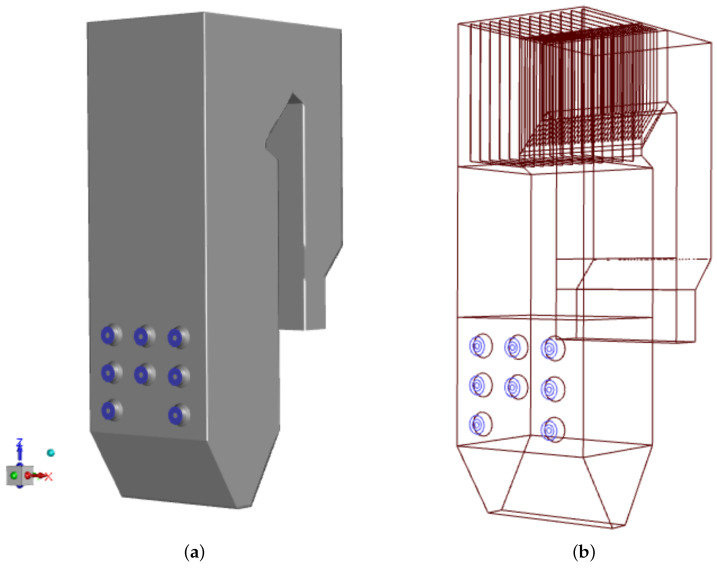
Geometrical model of the studied OP-230 type boiler: the general view with the front wall showing the burners’ system (blue circles) (**a**) and the first-pass details with superheater (**b**).

The reason for the study was the rapid sulfur corrosion that appeared in the OP-230 boiler after the low-swirl burners were installed. We chose to compare thermal, flow, and chemical conditions in the boiler operated with two types of burners, which differ significantly, i.e., the low-swirl burner (LSB) and the classic type, high-swirl burner (HSB). The authors had long-term measurement data on temperature, exhaust gas composition, and mass loss rates on exchanger tube walls for the boiler operated using these two types of burners. The measurement campaigns were carried out on a real-scale boiler operating in the Polish heat and power plant. The geometrical model of the case-study boiler, reflecting the original OP-230 boiler (scale 1:1) is displayed in Figure 1. The height of the analyzed unit is 30 m, the width of its combustion chamber is 8.4 m and its depth is 6.6 m. The external diameter of the swirl burner is 1 m.

Numerical modeling of pulverized coal combustion in the OP-230 boiler combustion chamber is based on modeling the flow and chemical reactions of gas in an Eulerian reference frame and modeling the movement of coal particles in a Lagrangian reference frame. The simulations aimed to determine whether there is a correlation between the rate of sulfur corrosion and the environment in the combustion chamber. For this purpose, the computations of the pulverized coal combustion process in two burner types were performed using ANSYS Fluent. To calculate the corrosion index (CI), the User-Defined Function (UDF) extension was utilized, and custom functions were written in the C programming language. Furthermore, to model the chemistry of fuel combustion, the mixture fraction approach is utilized. The mixture fraction represents the mass fraction of the fuel in a fuel/oxidizer mixture. This approach simplifies the tracking of species transport by reducing it to a single scalar. Through this assumption, the computational time needed to solve all necessary transport equations for each chemical element involved in each single-step chemical reaction is significantly shortened. The combustion process is parameterized by the mixture fraction and its variance, and chemical reactions are retrieved from pre-tabulated data. Thus, the software solves a transport equation for the mixture fraction and the variance in the mixture fraction, which accounts for the effects of turbulence in the mixing process.

A three-dimensional structured numerical mesh was applied. Through a grid independence analysis, an optimal structure composed of 1,400,000 cells in total, with improved mesh in the area of burners, was selected to provide a compromise between computational efficiency and accuracy. The results obtained constituted the basis of a numerical evaluation of the corrosion conditions of the boiler tube walls by calculating the CI function. Combustion modeling was carried out for typical operating conditions when six burners (i.e., those in the outermost rows) were in operation. The fuel mixing degree function *f* under non-adiabatic conditions was used in the process simulations. The fuel composition (on a dry ash basis (daf)) used in computations was adopted on the basis of the boiler operating data (Table 1). For the defined fuel, a PDF module was created; it contains chemical tables of the mixture compositions depending on the degree of mixing *f* and its variance $f^{\prime 2}$.

Fuel was supplied at a rate of 1.2 kg/s per burner. Based on our own measurements and the manufacturer’s data, it was assumed for both cases that the air entering the combustion chamber separated in the burner into the core, primary, and secondary air in the following proportions: 5%, 35%, and 60%, respectively. The total airflow from all six burners was 192,000 ${\mathrm{Nm}}^{3}/h$. In the tests performed on a cold burner, a turbulence intensity of 10% and a characteristic dimension of turbulence scales equal to 1 cm were measured. The primary air, where pulverized coal also flows, had a higher turbulence intensity (20%) and larger vortex scales (2 cm), while the secondary air had a slightly less turbulent flow (with the intensity of 15%) with a vortex scale similar to that observed for the primary air. The main parameter that differs between the two burner types is the degree of swirl of the air and air–fuel mixture. For high-swirl burners (HSBs), it was assumed that the tangential component of the velocity vector for the primary air is 0.4 of the velocity magnitude, and it is 0.45 for the secondary air. In the case of core air, the stream outflows normally to the inlet surface without a swirl. Based on the measurements presented in [47] it was determined that for low-swirl burners (LSBs), the tangential component of the velocity vector in the primary air region was 0.2 of the total velocity magnitude, whereas for the secondary air it was 0.225.

**Figure 2:**
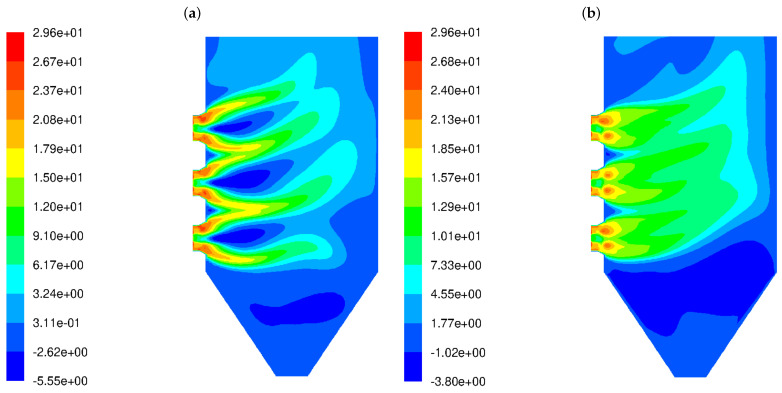
Isolines of horizontal component of the gas velocity vector (m/s) in the OP-230 boiler operated with high-swirl burners (**a**) and low-swirl burners (**b**).

Figure 2 shows the distribution of the horizontal component of the velocity vector for HSBs (Figure 2a) and LSBs (Figure 2b). In the figure displaying the flow predictions for the HSB (Figure 2a), the backflow areas near the burner outlet are clearly visible; these are represented by the negative values of a horizontal component of the velocity vector. The simulation results indicate a short outlet gas flow, which is further dispersed due to strong turbulence. In the case of the LS burner type (Figure 2b), there are no backflows around the burners and the gas outflow range is larger. The high-velocity gas streams deeply penetrate the combustion chamber.

**Figure 3:**
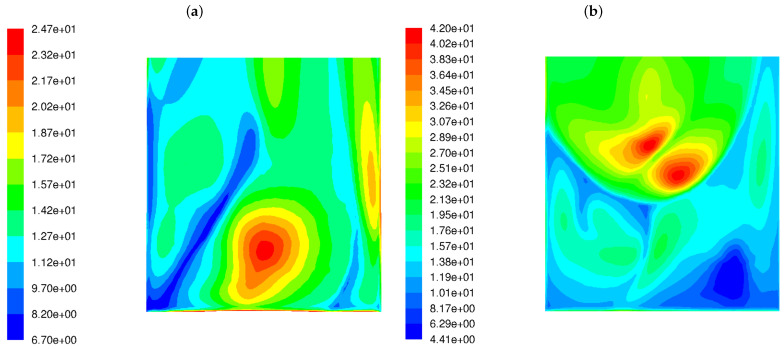
Frequency of the turbulent velocity fluctuations $\omega_{vel}$ in the wall area near the rear tube screens in the OP-230 boiler operated with high-swirl burners (**a**) and low-swirl burners (**b**).

Our analysis involved the distributions of various flow and chemical parameters at a distance of 5 cm from the rear wall across the boiler width, between 8 m and 18 m of the boiler height. The frequency of velocity field fluctuations $\omega_{vel}$ takes into account the dynamics of the process (changes in the fluid velocity field), as well as another parameter called mixture fraction variance ($f{’}^{2}$) that shows the dynamics of the chemical compounds’ concentration. Since simplified geometry does not reflect the original tube walls of the boiler, it was decided to present the flow parameters at a distance of 5 cm from the external walls (as the diameter of the heating exchanger pipes is 10 cm). Those parameters are involved in the proposed CI parameter (see Equation (11)). Velocity fluctuation $\omega_{vel}$ refers to the variations in velocity from its average or mean value over time or space. In turbulence, fluid particles move in random, chaotic patterns, causing rapid changes in velocity. Thus, velocity fluctuations are important for understanding how energy is distributed in turbulent systems. Higher velocity fluctuations are typically associated with more intense turbulence. When velocity fluctuations are high, it means the flow is highly unstable, with eddies, swirls, and rapid changes in the direction and magnitude of the velocity. High turbulence intensity can increase the mixing and transport of heat, mass, and momentum, which is very important in reactive flows. Figure 3 shows the distributions of $\omega_{vel}$ magnitudes in the wall area near the left and rear tube screens for the HS and LS burners. The difference in the frequency level of velocity fluctuations is visible. The higher level of $\omega_{vel}$ in the case of the LS burner means that the mixing process is not only more dynamic but also influences the other (in comparison to HS burner) chemical species concentration. In the high-swirl case, the maximum frequency is 24.7 1/s, while in the low-swirl case, it increases significantly to 42 1/s. It should be emphasized that the levels and scales of turbulence at the outlet of the burners were assumed to be the same.

**Figure 4:**
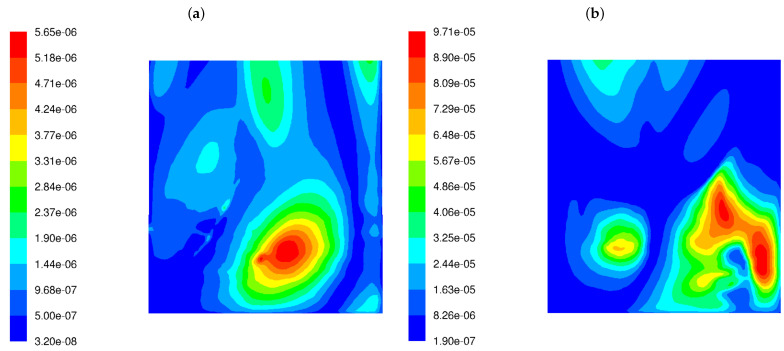
Distribution of the variance of the mixing degree $f^{\prime 2}$ in the OP-230 boiler operated with high-swirl burners (**a**) and low-swirl burners (**b**).

The variance of the mixing degree $f^{\prime 2}$ near the rear wall for both burner types is displayed in Figure 4. It can be seen from the figure that LS burners cause a much higher variance in the flue gas chemical composition near the rear tube screen wall. The level of variance in the mixing degree predicted for the LSBs is an order of magnitude greater than for the classic burners.

**Figure 5:**
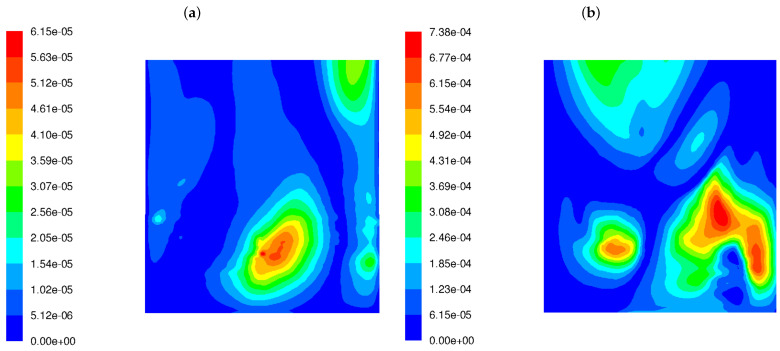
Distribution of the corrosion index (CI) in the OP-230 boiler operated with high-swirl burners (**a**) and low-swirl burners (**b**).

Figure 5 demonstrates the distribution of the CI function, described by Equation (7), over the whole width of the rear tube screen for both burners. The lower turbulence of the fuel–air mixture at the combustion chamber inlet causes the CI to increase by an order of magnitude on the rear screen. The simultaneous presence of factors such as the higher fluctuations in the exhaust gas composition and the higher frequencies of a velocity field disturbance results in an increase in corrosion index. Figure 5 shows that the corrosion hazard estimated by the proposed CI function has increased by a factor of 12.

Table 2 summarises the maximum values obtained from the simulation, including temperature, CO content, velocity fluctuation frequency, the variance of mixing degree, and the corrosivity index (corrosion probability) CI near the back screen tube wall. In the last row of the table, the corrosion rates are presented; these were derived from the long-term pipe thickness measurements in a boiler of the type considered in this study.

It may be concluded that calculated flow and chemical parameters are much higher for LS burners than for HS burners, and this is particularly pronounced for $f^{\prime 2}$ and CI. These parameters’ values appear to be several times higher for the case when the LSBs are under operation, as demonstrated by the following equation:

(12) $$f_{\mathrm{LSB}}^{\prime 2}/f_{\mathrm{HSB}}^{\prime 2}=17.0,$$

(13) $${\left(CI\right)}_{\mathrm{LSB}}/{\left(CI\right)}_{\mathrm{HSB}}=11.9.$$

These coefficients indicate that the use of burners with a lower degree of swirling transmits larger fluctuations of chemical processes combined with more frequent oscillations in the flow structure towards the rear tube screen wall. The obtained numerical results of coal dust combustion in a pulverized coal boiler show qualitative agreement between the predictions of the proposed corrosion development model and the corrosion rate measurements. The corrosion rate of the rear wall with the use of LS burners is almost eight times higher than it is when HS burners are used.

## 5. Summary and Conclusions

The commonly used models of corrosion have specific applications depending on whether the investigation is targeted at predicting the extent or the rate of corrosion or at understanding material behavior under a combination of mechanical and chemical stress. Nevertheless, they are focused on predicting the detailed processes occurring mostly in a solid–fluid interaction zone.

In this work, authors proposed a new approach that can be implemented for any CFDs solver. The study revealed that CFDs can be used not only for flow and heat transfer analysis but may also serve to assess the effects of electrochemical processes in the material. The developed model does not take into account electrochemical phenomena occurring on the surface of the boiler tubes, but only the flow and chemical processes in the flue gas near the boiler walls. The proposed coefficient of chemical and flow conditions is not a measure of corrosion per se, but a factor that determines the possibility of corrosion. The essence of the proposed approach is to simplify the description of corrosion and limit it only to factors originating from the flue gas environment characteristics, without going into the detailed description of microscale processes on the tube surface. In addition to CO concentration, it utilises the frequency of velocity field fluctuations and the mixture fraction variance to determine the areas at risk of corrosion.

The modelling outcomes regarding the flue gas composition and temperature and velocity fields for a 250 MW pulverized fuel boiler show fundamental differences between the two very different types of burners. The numerically obtained results indicate that the conditions in the boiler operated with burners of low air–fuel mixture swirling generate conditions conducive to the development of corrosion. This is consistent with the outcome of the long-term corrosion rate measurement campaign in the studied large-scale boiler type.

## Figures and Tables

**Table 1:** Coal composition (wt.) assumed in calculations (daf basis).

Component	*Y*, kg/kg
Carbon	0.82
Hydrogen	0.05
Oxygen	0.11
Sulfur	0.01
Nitrogen	0.01

**Table 2:** Maximum parameter values obtained for different type of burners.

	HSB	LSB
Temperature, °C	800	920
CO, %	2.0	4.3
$\omega_{vel}$, 1/s	25	42
$f^{\prime 2}$, $\times 10^{-6}$	5.7	97.1
CI, $\times 10^{-5}$	6.2	73.8
Corrosion rate **, mm/year	0.09	0.7

** derived from long-term measurements.

## Data Availability

The original contributions presented in the study are included in the article, further inquiries can be directed to the corresponding author.

## Nomenclature

*A*	Surface area, $m^{2}$
CI	Corrosion index, -
*f*	Mixture fraction, -
$f^{\prime }$	Variance of mixture fraction, -
*k*	Turbulence kinetic energy, J/kg
$k_{c}$	Reaction rate constant, 1/s
*T*	Temperature, K
Greek symbols	
ε	Turbulence kinetic energy rate, $m^{2}/s^{3}$
$\omega_{chem}$	Frequency of redox reactions fluctuations, 1/s
$\omega_{vel}$	Frequency of velocity field fluctuations, 1/s
Subscripts	
*d*	Turbulence kinetic energy rate, $m^{2}/s^{3}$
*m*	Melting
ox	Oxidation
*r*	Reduction
Subscripts	
in	Inlet
out	Outlet
st	Stack
*	Adiabatic combustion
Abbreviations	
CFB	Coal fluidized bed
daf	Dry ash-free
HSB	High-swirl burner
LSB	Low-swirl burner
MSW	Municipal solid waste
